# YOLO-RD: A Road Damage Detection Method for Effective Pavement Maintenance

**DOI:** 10.3390/s25051442

**Published:** 2025-02-27

**Authors:** Wei Wang, Xiaoru Yu, Bin Jing, Ziqi Tang, Wei Zhang, Shengyu Wang, Yao Xiao, Shu Li, Liping Yang

**Affiliations:** 1 College of Computer Science and Technology, Changchun University, No. 6543, Satellite Road, Changchun 130022, China; 231501508@mails.ccu.edu.cn (X.Y.); 231502527@mails.ccu.edu.cn (B.J.); 231502540@mails.ccu.edu.cn (W.Z.); 231502533@mails.ccu.edu.cn (S.W.); 241503545@mails.ccu.edu.cn (Y.X.); 241501501@mails.ccu.edu.cn (S.L.); yangliping@ccu.edu.cn (L.Y.); 2 School of Construction Engineering, Jilin University, No. 2699, Qianjin Street, Changchun 130012, China; tangzq2424@jlu.edu.cn

**Keywords:** road damage detection, YOLO-RD, star operation module, multi-dimensional auxiliary fusion, wavelet transform convolution

## Abstract

Road damage detection is crucial for ensuring road safety and minimizing maintenance costs. However, detecting small damage, managing complex backgrounds, and identifying irregular damage shapes remain significant challenges. To address these issues, we propose YOLO-RD, an advanced detection framework that integrates innovative modules for feature enhancement, multi-scale robustness, and detail preservation. Specifically, the Star Operation Module (SOM) improves sensitivity to small-scale damage, the Multi-dimensional Auxiliary Fusion (MAF) module strengthens robustness in complex environments, and the Wavelet Transform Convolution (WTC) enables adaptive focus on irregular shapes. On the Japanese road dataset in RDD2022, YOLO-RD achieves a detection accuracy of 25.75%, with a notable 4.93% improvement in small object detection over the baseline YOLOv8. These results demonstrate the effectiveness and practicality of YOLO-RD in addressing diverse and challenging real-world scenarios, establishing it as a robust solution for automated road condition monitoring.

## 1. Introduction

Road damage, a common type of road degradation, severely impacts the lifespan of roads. With over 5.44 million kilometers of highways in China, which handle enormous traffic volumes each year, issues such as road cracks and potholes emerge frequently, exacerbated by changing climatic conditions [1]. These problems pose risks to road safety and increase maintenance burdens, making road damage detection essential [2]. Traditional manual inspection methods rely on on-site checks and data recording, which are both time-consuming and labor-intensive. Furthermore, the results are easily influenced by subjective factors, leading to unstable outcomes. With the development of computer vision and deep learning, automated detection technology offers an efficient, accurate, and cost-effective solution that enables the rapid processing of large-scale road damage images [3]. However, road damage detection faces three major technical challenges. First, small object detection [4] is challenging, as fine cracks are easily overlooked in large-scale images, leading to reduced recognition accuracy. Second, complex background [5] interference, including lighting changes, stains, and rain, can affect the discrimination of the model, causing false detections. Lastly, irregular damage shapes [6], such as irregular cracks and potholes, make it difficult to apply fixed rules for effective recognition. These issues complicate feature extraction and pattern recognition, increasing the difficulty of model optimization. Therefore, the discussion and research presented in this paper hold significant practical importance.

In the field of road damage detection, traditional methods such as laser detection [7] and geomagnetic detection [8] are overly reliant on manual labor, resulting in poor detection performance. These methods are unable to pinpoint the exact location of damage, and they are costly and require complex maintenance. Rule-based image processing methods [9], although they can improve detection efficiency to some extent, primarily rely on predefined feature extraction algorithms. They lack the ability to adapt to changes in environmental lighting and complex backgrounds, making it difficult to handle the diverse damage shapes encountered in real-world scenarios. In contrast, data-driven road damage detection methods [10], which use deep learning models to automatically learn and extract features from images, can accurately localize and identify damage. Zhang et al. [11] proposed a fast pavement damage detection network (FPDDN) for real-time and high-accuracy road damage detection. Ren et al. [12] proposed a novel road damage detection method based on an improved YOLOv5 network and street view images. This model can handle large-scale detection layers to enhance the detection accuracy of large damaged objects. These methods demonstrate significant advantages in terms of adaptability and robustness, reducing the influence of human subjectivity. Furthermore, through continuous model optimization and data updates, they effectively respond to varying damage shapes and environmental changes.

Object detection [13] is a fundamental task in computer vision, and significant progress has been made in research on object detection in recent years. Numerous studies have developed various efficient object detection methods, which not only continuously improve accuracy but also optimize processing speed and model lightweight design. For example, the You Only Look Once (YOLO) [14] series models achieve real-time detection with their single-stage structure, making them suitable for scenarios that require quick responses. Two-stage detection models, such as Mask R-CNN [15], offer better accuracy and are commonly used in applications that demand high detection precision. Additionally, models like Efficientnet [16] and Single Shot MultiBox Detector (SSD) [17] combine multi-scale feature fusion and lightweight design, balancing both detection efficiency and accuracy, thus laying a solid foundation for the wide application of object detection in practical scenarios. In this study, object detection methods are widely applied to road damage detection tasks. These methods can quickly and accurately identify and localize damage regions while effectively handling variations in damage shapes and sizes. Although segmentation methods provide pixel-level damage information, they have higher computational costs and slower processing speeds. Object detection, while maintaining high accuracy, offers faster processing speeds, significantly enhancing the practicality and reliability of detection systems.

**Figure 1:**
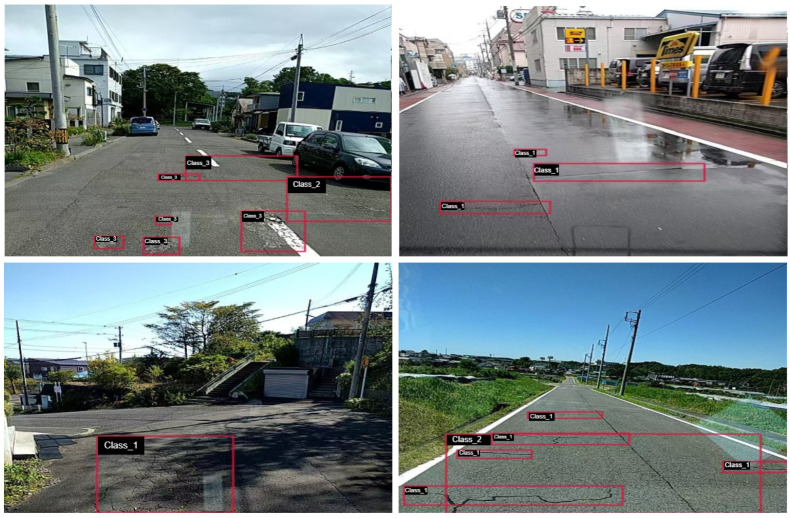
Visualization of road damage detection samples from the real world.

The following details the three existing challenges and their impacts: as shown in Figure 1, there are four categories (class_0, class_1, class_2, class_3), which, respectively, represent longitudinal cracks, transverse cracks, alligator cracks, and potholes. These challenges include small object detection, complex background interference, and the damage of irregular shapes. Small object detection is particularly challenging in road damage recognition, especially for fine cracks. Due to their small size, crack features are often not prominent in images, especially in high-resolution images, where the edges of cracks may be difficult to distinguish due to blurring or noise. The low contrast between small cracks and the surrounding road surface further increases the difficulty for the model, leading to false positives or missed detections. Additionally, because small damage occupies a small percentage of the pixels, it can be overlooked during global feature extraction, which is a problem that is exacerbated in low-resolution or noisy images [18]. Complex background interference is another significant challenge in road damage detection. Common environmental factors, such as changes in lighting angles, tire tracks, and water coverage, often resemble or interfere with damage patterns, making them difficult to accurately identify. This background noise causes the model to misclassify the background as a damage site or ignore actual damage, thereby affecting detection accuracy. Furthermore, background interference increases the difficulty of feature extraction, further lowering the reliability and efficiency of the detection system [19]. Irregular damage shapes pose an additional challenge for feature extraction. Cracks are influenced by various factors during formation, resulting in discontinuous, branching, or mesh-like structures. These irregular shapes make it difficult for the model to extract consistent features, as feature information may be blurred or lost, especially during the deeper stages of feature extraction. The complexity of damage shapes further exacerbates the difficulty of model in capturing fine details, impacting the accuracy and robustness of detection [20].

To address the aforementioned challenges, we propose an improved detection framework based on YOLOv8 [21]. First, a Star Operation Module (SOM) [22] is integrated into the third and fourth stages of the backbone network. By projecting features into a high-dimensional space, this module retains the feature extraction capability for small objects (such as fine road cracks), thereby enhancing the performance of model in detecting small objects. Next, a Multi-dimensional Auxiliary Fusion (MAF) [23] module is introduced in the neck of the network. This module incorporates the multi-scale and Attention Refinement Module (ARM) to further fuse features from multiple dimensions, enhancing the representation ability of the features and improving the robustness of feature representation in complex backgrounds. Finally, Wavelet Transform Convolution (WTC) [24] replaces traditional convolution in the head of the network. By using wavelet transforms to decompose the input features into multi-frequency components, this method effectively expands the receptive field and focuses on low-frequency shape information, thereby improving the ability of model to capture irregular damage patterns.

To briefly summarize our contributions, we present them in five key aspects:

(1) We propose a novel road damage detection model that combines the Star Operation Module (SOM), Multi-dimensional Auxiliary Fusion (MAF) module, and Wavelet Transform Convolution (WTC) module to address the challenges of detecting road damage in complex scenarios.

(2) To improve detection of small-scale damage, such as fine cracks, we introduce the SOM. This module enhances the representation of small object features by utilizing high-dimensional feature projections.

(3) To tackle the challenges posed by complex backgrounds and feature interference, we propose the MAF module, which incorporates an Attention Refinement Module (ARM) to enhance feature robustness and discriminative power.

(4) To handle irregular damage shapes, we introduce the WTC, which decomposes input features into multi-frequency components, expanding the receptive field and improving the ability of model to capture low-frequency shape information.

(5) The proposed method was validated on public road damage datasets, achieving an average accuracy of 25.75%, making it a powerful and efficient solution for road damage detection in complex environments.

## 2. Related Work

### 2.1. One-Stage Detectors

One-stage object detectors, such as the YOLO and SSD series, prioritize real-time performance by predicting object classes and bounding box coordinates in a single forward pass, bypassing the region proposal step used in two-stage detectors. This architecture makes one-stage detectors highly efficient for real-time applications like autonomous driving and video surveillance. Single Shot MultiBox Detector (SSD) [17] leverages multi-scale feature maps and default anchor boxes to balance detection speed and accuracy, making it suitable for applications requiring efficient and lightweight solutions. YOLOv4 [25] introduced CSPNet for optimized feature learning. YOLOv6 [26] further improved performance by incorporating re-parameterizable backbones and Rep-PAN neck designs, boosting speed and accuracy. YOLOv8 brought additional advancements, including anchor-free detection mechanisms, dynamic task prioritization, and architectural refinements for robust and flexible real-time performance. YOLOv10 [27] took the field a step further by eliminating the non-maximum suppression (NMS) process, replacing it with a consistent dual assignment approach, ensuring both low latency and reliable detection. However, despite their speed advantage, one-stage detectors often face challenges in detecting small objects or handling complex backgrounds due to the absence of a refined region proposal mechanism.

### 2.2. Two-Stage Detectors

Two-stage detectors are designed to provide high accuracy, particularly in tasks that require precise object localization. R-CNN [28] introduced the use of selective search for region proposals and CNNs for classification and bounding box regression, but it was computationally inefficient due to redundant feature extraction. Fast R-CNN [29] improved upon this by using shared convolutional feature maps and RoI pooling, speeding up the process, though it still relied on external region proposal generation. Faster R-CNN [30] further advanced the model by introducing the Region Proposal Network (RPN), allowing the network to generate proposals internally and making the entire pipeline end-to-end, thus improving both speed and accuracy. Mask R-CNN [31] extended Faster R-CNN by adding a segmentation branch for pixel-level object masks, making it suitable for instance segmentation but increasing computational cost. Cascade R-CNN [32] refined proposals across multiple stages with progressively higher IoU thresholds, enhancing detection precision, especially for challenging objects, but at the cost of added computational complexity. While two-stage detectors excel in accuracy, their computational demands often make them less suitable for real-time applications compared to one-stage models.

### 2.3. Similar Works

Many researchers have already similar work on solving the problem of road damage detection [33,34,35]. Du et al. [36] propose a lightweight object detection algorithm with enhanced feature extraction based on the You Only Look Once (YOLO) algorithm. The Bidirectional Feature Pyramid Network (BIFPN) network structure is used for multi-scale feature fusion to enhance the feature extraction ability, and Varifocal Loss is used to optimize the sample imbalance problem, which improves the detection accuracy of road damage. Li et al. [37] introduce a novel approach for identifying and locating road damage based on an enhanced ML-YOLO algorithm. By refining the YOLOv8 object detection framework, they optimize both the traditional convolutional layers and the spatial pyramid pooling network. Despite significant advancements in road damage detection, many challenges remain, particularly in detecting small cracks and handling complex road backgrounds. These issues arise from the difficulty of detecting small objects and the need for robust feature representation to deal with diverse and irregular damage shapes. As a response, we propose the YOLO-RD model, which builds upon these existing approaches and introduces novel components to address the aforementioned challenges.

Starting with Feature Pyramid Networks (FPNs) [38], feature fusion strategies in object detection have undergone several advancements. An FPN uses a top-down pathway with lateral connections to pass high-level semantic information to lower layers, enhancing multi-scale detection. Later, the Asymptotic Feature Pyramid Network (AFPN) [39] introduced Asymptotic Feature Fusion (AFF), which progressively guides feature fusion to optimize multi-scale information interaction. Path Aggregation Networks (PANets) [40] extended FPNs by adding a bottom-up path augmentation, strengthening information flow from low-level features. CM-Net [41] focused on arbitrary-shaped text detection with a concentric feature fusion strategy for better local–global feature representation. Finally, EfficientDet [42] proposed the Bidirectional Feature Pyramid Network (BiFPN), which adaptively learns the importance of different feature levels using weighted connections, improving efficiency and detection accuracy. In contrast, our Multi-dimensional Auxiliary Fusion (MAF) module goes beyond traditional feature fusion strategies by introducing a more flexible and adaptive approach to integrate information across different feature dimensions. Unlike the static hierarchical fusion seen in FPN or BiFPN, the MAF module employs an Attention Refinement Module (ARM) to dynamically refine feature maps at different scales, ensuring that the most relevant features are emphasized while less important ones are suppressed. This adaptive fusion process enhances feature robustness and discriminability, which is particularly crucial for detecting small cracks and handling complex backgrounds in road damage detection.

Furthermore, the Star Operation Module (SOM) is designed to specifically address the challenge of small object detection. SOM enhances the representation of small object features by leveraging high-dimensional feature projections. It achieves this by utilizing star-shaped convolutions that capture fine-grained details, which are often overlooked in traditional convolutional layers. This module helps improve the detection of small road cracks, which are typically difficult for conventional methods to accurately detect. Additionally, to tackle the issue of irregular damage shapes, we introduce the Wavelet Transform Convolution (WTC) module, which decomposes input features into multi-frequency components, expanding the receptive field and improving the model’s ability to capture low-frequency shape information.

## 3. Methodology

### 3.1. Architecture of the Proposed Method

**Figure 2:**
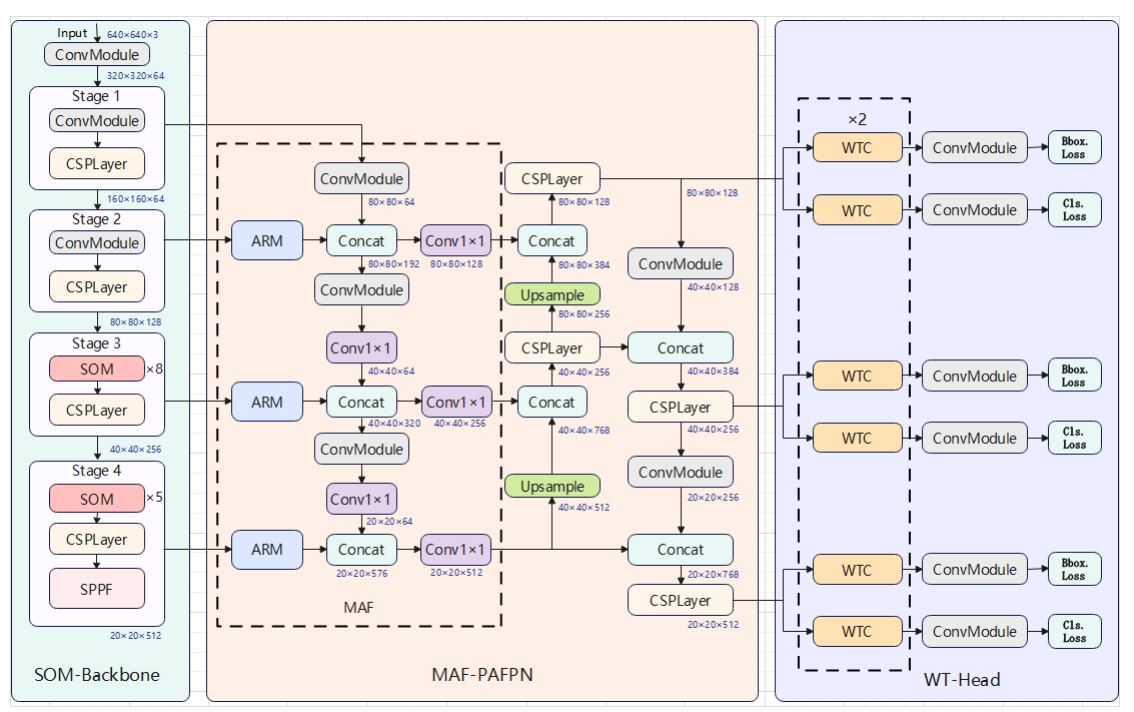
Architecture of the proposed method.

To address the challenges of road damage detection, including detection of small objects, complex background interference, and irregular damage shapes, we propose YOLO-RD, inspired by the architecture of YOLOv8. As shown in Figure 2, the overall architecture of YOLO-RD highlights the key components and modifications designed to tackle these challenges. While the original YOLOv8 model excels in general object detection tasks, it faces difficulties in meeting the specific demands of road damage detection. To address these limitations, YOLO-RD introduces several critical innovations, including the Star Operation Module (SOM) [22], Multi-dimensional Auxiliary Fusion (MAF), Wavelet Transform Convolution (WTC) [24], and Attention Refinement Module (ARM) [23]. These enhancements aim to improve the robustness of model and accuracy in road damage detection. The YOLO-RD framework comprises three main components: SOM-Backbone, MAF-PAFPN, and WT-Head.

In the YOLO-RD model, the features are first input into the backbone network, where they are subjected to four stages of feature extraction operations to obtain four output features. These four outputs are the inputs to the detection neck. In the detection neck, the features are first processed by MAF, and the four inputs are first processed by the ARM, and then they are spliced and adjusted from top to bottom through a series of channel dimensions, followed by a bottom-up and top-down feature fusion process. The detection neck outputs three features, which are also used as the input of the detection head. In the detection head, they are subjected to Wavelet Transform Convolution, and then classified and regressed, respectively.

The SOM-Backbone is an improved version of the YOLOv8 Backbone (CSPDarknet), which is designed to enhance small object detection in road damage detection tasks. CSPDarknet extracts low-level features such as edges, textures, and gradients through a split-and-merge strategy, reducing redundancy and improving computational efficiency. However, it has limitations in detecting small objects, as it struggles to retain fine-grained details, which affects the accuracy of road damage detection. To address this, we propose the SOM-Backbone. By applying element-wise multiplication, the Star Operation Module (SOM) enhances the representation of small-scale features, significantly improving the sensitivity of the network to minor road damage.

MAF-PAFPN, an enhanced version of PAFPN, addresses challenges in road damage detection by incorporating the Multi-dimensional Auxiliary Fusion (MAF) module. While PAFPN performs well in general object detection tasks, it faces significant challenges in road damage detection due to the complex and variable nature of road surfaces, which are often cluttered with occlusions and varying textures. MAF-PAFPN combines low-level spatial features with high-level semantic information through multi-scale feature aggregation and the Attention Refinement Module (ARM). The ARM refines feature maps by emphasizing critical regions, while multi-scale aggregation captures fine details across scales. This fusion improves robustness in cluttered environments and enhances detection accuracy for small road damage, such as cracks and potholes, in complex real-world conditions.

The Wavelet Transform Head (WT-Head) is the detection head of YOLO-RD, which is responsible for final object classification and bounding box regression. It processes features from the backbone and neck to predict object categories and locations. In road damage detection, especially for small and irregular cracks, the standard YOLOv8 head struggles with accurate detection and localization. To overcome this, WT-Head integrates Wavelet Transform Convolution (WTC), which enhances multi-scale feature extraction by decomposing feature maps into distinct frequency components. This enables the model to capture both fine details and broader structural patterns, improving detection accuracy for subtle road damage. In addition, WTC gradually reduces the receptive field of features in the process of using wavelet transform and inverse wavelet transform, and uses smaller convolution kernels for convolution operations, thereby reducing computational costs and promoting lightweight design of the model.

### 3.2. Star Operation Module

**Figure 3:**
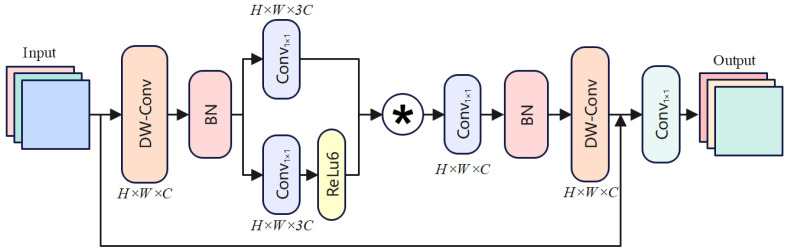
Star Operation Module.

As illustrated in Figure 3, to address the challenge of detecting small cracks in road damage detection, we propose the Star Operation Module (SOM), a novel nonlinear feature fusion method. The SOM enhances the representation of small-scale features while preserving high-level semantic information, ensuring high detection accuracy. The module integrates depthwise convolutions, pointwise convolutions, element-wise multiplication, nonlinear activations, residual connections, and a final 1×1 convolution to refine feature maps effectively. The design concept of the SOM is analogous to stars in the universe, gradually bringing distant objects closer and focusing on small features. Imagine small cracks in an image as thin lines, easily drowned out by background noise. The * operation shown in Figure 3 represents element-by-element multiplication. The SOM extracts spatial details and semantic information of the cracks through two branches, and then dynamically fuses these features using element-wise multiplication, much like using a magnifying glass to focus on the crack area, making its features more prominent. Finally, through residual connections, the original feature information is preserved, ensuring that the details of the cracks are not lost in the deeper layers of the network. The transformation in SOM can be expressed as follows:

(1) $$F_{\mathrm{fused}}={\mathrm{Conv}}_{1\times 1}\left(\mathrm{BN}\left(\mathrm{DWConv}1\left(F_{\mathrm{input}}\right)\right)\right)⊙\mathrm{ReLU}6\left({\mathrm{Conv}}_{1\times 1}\left(\mathrm{BN}\left(\mathrm{DWConv}1\left(F_{\mathrm{input}}\right)\right)\right)\right)$$

(2) $$F_{\mathrm{intermediate}}=\mathrm{DWConv}2\left(\mathrm{BN}\left({\mathrm{Conv}}_{1\times 1}\left(F_{\mathrm{fused}}\right)\right)\right)+F_{\mathrm{input}}$$

(3) $$F_{\mathrm{output}}={\mathrm{Conv}}_{1\times 1}\left(F_{\mathrm{intermediate}}\right)$$

where $F_{\mathrm{input}}$ represents the input feature map. $F_{\mathrm{fused}}$ represents the fused feature map, which is obtained by applying depthwise convolutions and pointwise convolutions, followed by element-wise multiplication and ReLU activation. This map enhances the spatial and semantic representation of small-scale features. $F_{\mathrm{intermediate}}$ is the intermediate feature map after further refinement using depthwise convolution and residual addition with the original input features. Assume the input feature has dimensions H×W×C, where *H* and *W* denote the spatial dimensions and *C* is the number of channels. The input feature undergoes a series of transformations to enhance its representation while preserving spatial and semantic details. Firstly, the input feature map is processed by a Depthwise Convolution (DWConv1) to extract spatial features independently for each channel. The resulting feature map retains the same spatial dimensions H×W and channel count *C*. Next, Batch Normalization (BN) is applied to stabilize the mean and variance of the feature map, ensuring consistent training. The normalized feature map dimensions remain H×W×C. Following normalization, the feature map is divided into two branches. The first branch applies a Pointwise Convolution (${\mathrm{Conv}}_{1\times 1}$), adjusting the channel count to 3C. The second branch undergoes the same transformation, followed by a ReLU6 activation to introduce nonlinearity by clamping values to the range [0, 6]. The outputs of both branches are fused via element-wise multiplication (⊙), producing the enhanced feature map $F_{\mathrm{fused}}$ with dimensions H×W×3C. Subsequently, the fused feature map passes through another Pointwise Convolution (${\mathrm{Conv}}_{1\times 1}$), reducing the channel count back to *C*. This is followed by a Depthwise Convolution (DWConv2), which refines spatial features. The resulting feature map $F_{\mathrm{intermediate}}$ retains dimensions H×W×C. To preserve the original spatial and semantic information, a residual connection adds $F_{\mathrm{input}}$ to $F_{\mathrm{intermediate}}$. Finally, the enhanced feature map is refined through a last Pointwise Convolution (${\mathrm{Conv}}_{1\times 1}$), adjusting the channel dimensions to $C^{\prime }$. This produces the final output feature map $F_{\mathrm{output}}$ with dimensions $H\times W\times C^{\prime }$.

### 3.3. Multi-Dimensional Auxiliary Fusion

To tackle the challenge of small damage detection in road damage detection, especially in the presence of complex backgrounds, we propose the Multi-dimensional Auxiliary Fusion (MAF) module, integrated with an Attention Refinement Module (ARM). This innovation introduces an advanced strategy for feature fusion and dynamic attention adjustment, allowing the model to better focus on important features, such as small cracks, while suppressing irrelevant background information. As illustrated in Figure 2, the MAF module begins the fusion process early in the network, combining both low-level spatial features and high-level semantic features. By doing so, it ensures that crucial fine-grained details, which are essential for detecting small damage, are preserved throughout the network. The fusion process between different stages helps integrate multi-scale information, making it more effective for detecting subtle road damage.

The MAF module integrates features from multiple stages, dynamically adjusting the contribution of each stage based on its relevance to the detection task. The feature fusion process can be mathematically expressed as follows:

(4) $$F_{\mathrm{MAF}}=\sum_{i=1}^{n}\alpha_{i}F_{{\mathrm{stage}}_{i}},\;\mathrm{where}\;\alpha_{i}\in R,\;\sum_{i=1}^{n}\alpha_{i}=1$$

where $F_{{\mathrm{stage}}_{i}}$ represents the feature map from each stage, and $\alpha_{i}$ is the dynamic weight assigned to the feature map of each stage. These weights are learned during training, allowing the model to adaptively prioritize more relevant features for crack detection. This process ensures that low-level and high-level features are fused to enhance the sensitivity of model to small damage.

While MAF focuses on aggregating multi-scale features, the ARM fine-tunes the feature maps generated at different stages of the network by applying spatial refinement to enhance key regions. The ARM works specifically after the output of Stage 2, Stage 3, and Stage 4 feature maps, improving their representation by dynamically focusing on critical regions such as small cracks, and reducing the impact of irrelevant background features.

**Figure 4:**
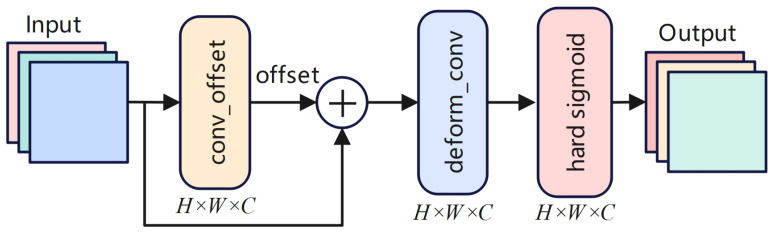
Multi-dimensional Auxiliary Fusion.

As shown in Figure 4, the Attention Refinement Module (ARM) plays a crucial role in refining feature maps through spatial feature adjustments to enhance the focus on critical regions such as small cracks, while suppressing irrelevant background features. ARM operates on feature maps generated from Stage 2, Stage 3, and Stage 4, where each stage contains feature maps with different levels of spatial and semantic information.

The ARM first generates spatial offsets Δpk via a convolution operation, adjusting the receptive fields of the network to focus on important regions, such as small cracks. This process is mathematically represented as follows:

(5) $$\Delta pk=\mathrm{conv}\_\mathrm{offset}(F_{{\mathrm{stage}}_{i}})$$

where $F_{{\mathrm{stage}}_{i}}$ represents the feature maps from different stages (Stage 2, Stage 3, Stage 4), and conv_offset is the convolution operation that generates the spatial offsets Δpk.

Next, the ARM applies deformable convolution using the generated offset Δpk, refining the feature maps to focus on discriminative regions while adjusting spatial locations. This process is mathematically expressed as follows:

(6) $$F_{\mathrm{refined}}=\mathrm{deform}\_\mathrm{conv}\left(F_{{\mathrm{stage}}_{i}}+\Delta pk\right)$$

where $F_{\mathrm{refined}}$ is the feature map after deformable convolution, and Δpk is the spatial offset generated in the previous step.

Following spatial refinement, the ARM further processes the adjusted feature map using a Sigmoid activation function to normalize the feature map, producing the final refined feature map $F_{\mathrm{ARM}}$. This step amplifies important regions (such as small cracks) and suppresses background noise, ensuring that key features are preserved. The final output is expressed as follows:

(7) $$F_{\mathrm{ARM}}=\mathrm{sigmoid}\left(\mathrm{deform}\_\mathrm{conv}\left(F_{{\mathrm{stage}}_{i}}+\Delta pk\right)\right)$$

where $F_{\mathrm{ARM}}$ represents the refined feature map, normalized using the Sigmoid function, with values constrained to the range [0, 1].

Finally, the refined feature map $F_{\mathrm{ARM}}$ is passed to the subsequent layers of the network for further processing. The MAF module effectively integrates multi-scale features from different stages of the network, ensuring the seamless fusion of low-level spatial features and high-level semantic features to retain critical details. Meanwhile, the ARM fine-tunes the feature maps at each stage, dynamically focusing on key regions associated with cracks and suppressing background noise. Together, these two modules address the challenges of detecting small cracks and handling diverse and irregular crack shapes in complex backgrounds. This synergy enhances the robustness and accuracy of model, providing an efficient and reliable solution for road crack detection under challenging real-world conditions.

### 3.4. Wavelet Transform Convolution

To improve the detection accuracy of irregular cracks in road damage detection, we propose the WT-Head, which modifies the original YOLOv8 detection head by replacing its standard convolution layers with the Wavelet Transform Convolution (WTC) module. As shown in Figure 2, the WT-Head consists of two main components: a classification head and a regression head. These components are supported by the WTC module, which replaces the standard convolutions in the detection head to enhance feature extraction by leveraging multi-frequency decomposition and processing.

**Figure 5:**
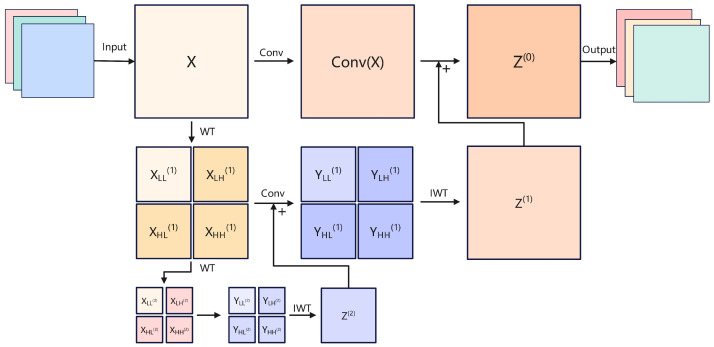
Wavelet Transform Convolution.

**Figure 6:**
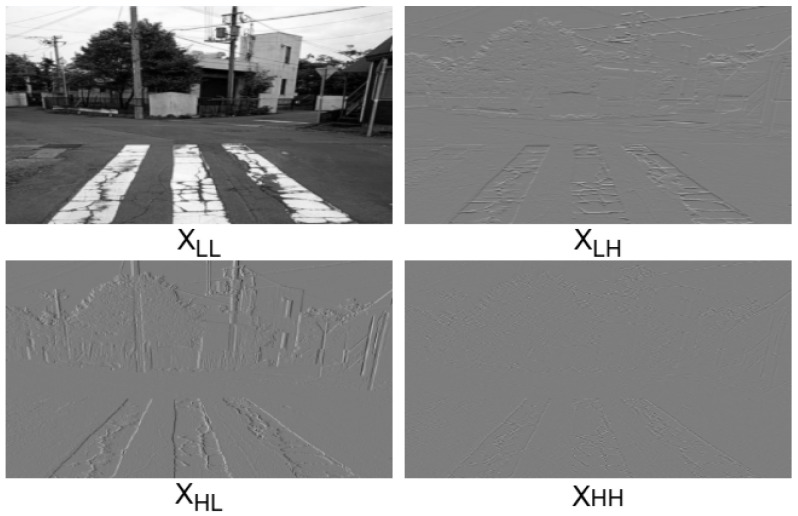
Frequency decomposition.

As shown in Figure 5, The WTC module begins by applying the wavelet transform (WT) to the input feature map *X*, splitting it into four frequency bands: $X_{LL}$ (low-frequency, capturing global structural patterns) and $X_{LH},\;X_{HL},\;\mathrm{and}\;X_{HH}$ (high-frequency components capturing horizontal, vertical, and diagonal edge details), as shown in Figure 6. The low-frequency component (LL) contains the main structure and contour information of the image, which is suitable for extracting the overall contour and location information of the crack, but may be interfered with by background texture and shadow. The horizontal high-frequency component (LH) captures the horizontal edge and detail information of the image, which is suitable for detecting transverse cracks. The vertical high-frequency component (HL) reflects the vertical edge and detail information in the image, which is suitable for detecting longitudinal cracks. The diagonal high-frequency component (HH) reveals the diagonal edge and detail information in the image, which is suitable for detecting potholes or irregular crack networks. By decomposing the image into these components, the crack features in different directions can be analyzed in detail. Combining the information of multiple components can not only effectively enhance the expression of crack features, but also suppress interference factors such as background noise, shadows, and stains. This multi-scale and multi-directional information fusion method can significantly improve the accuracy and robustness of road crack detection, especially in the cases of complex backgrounds and irregular shapes, and can more effectively identify and locate cracks.

This process is mathematically represented as follows:

(8) $$\left[X_{LL},X_{LH},X_{HL},X_{HH}\right]=WT\left(X\right)=\mathrm{Conv}\left(\left[f_{LL},f_{LH},f_{HL},f_{HH}\right],X\right)$$

where the Haar wavelet filters $f_{LL},\;f_{LH},\;f_{HL},\;f_{HH}$ are used to perform the decomposition. After decomposition, each frequency band is processed independently by small-kernel convolutions to extract features specific to each band. For example, $X_{LL}$ provides a smoothed, global representation, while $X_{LH},\;X_{HL},\;X_{HH}$ focus on high-frequency edge and texture information. To further expand the receptive field, the WTC module employs a hierarchical decomposition approach. The low-frequency component $X_{LL}$ undergoes recursive decomposition into multiple levels:

(9) $$X_{LL}^{\left(i\right)},X_{LH}^{\left(i\right)},X_{HL}^{\left(i\right)},X_{HH}^{\left(i\right)}=WT\left(X_{LL}^{\left(i-1\right)}\right)$$

where *i* represents the decomposition level. This process progressively increases the receptive field while retaining the spatial resolution of features. After convolutional operations are applied at each level, the outputs are recombined through the inverse wavelet transform (IWT):

(10) $$Z^{\left(i\right)}=IWT\left(Y_{LL}^{\left(i\right)}+Z^{\left(i+1\right)},Y_{LH}^{\left(i\right)},Y_{HL}^{\left(i\right)},Y_{HH}^{\left(i\right)}\right)$$

The final output $Z^{\left(0\right)}$ from the WTC module is seamlessly integrated into two distinct components for downstream processing. The Classification Head capitalizes on the low-frequency features ($X_{LL}$) to effectively discern regions containing cracks by leveraging the global structural context encoded within these features. Conversely, the Regression Head harnesses the high-frequency components ($X_{LH},\;X_{HL},\;X_{HH}$) to achieve precise delineation of crack boundaries, ensuring the accurate capture of intricate edge details and local textures. This dual-stream approach optimally exploits the hierarchical frequency information to balance global awareness with fine-grained localization.

Replacing the standard convolutions in YOLOv8 with WTC provides several advantages. The multi-frequency decomposition enables the model to efficiently separate and process both global and local information, improving detection performance on irregular cracks. The hierarchical decomposition significantly expands the receptive field without adding excessive computational overhead, ensuring both efficiency and accuracy. Moreover, the WTC module enhances the robustness of model against noise and varying background conditions, making it suitable for real-world road damage detection scenarios.

## 4. Experiment

### 4.1. Datasets and Evaluation Metrics

This paper utilizes the publicly available RDD2022 dataset [43], a multi-country collection of road images for automatic road damage detection, comprising 47,420 images from six countries: Japan, India, the Czech Republic, Norway, the United States, and China. After data cleaning, 7900 road images from Japan were selected for analysis. The Japanese dataset is particularly valuable for this study due to its diversity in both road types (e.g., urban and rural roads) and environmental conditions (e.g., sunny, rainy, and shaded conditions). These variations in rainfall and lighting angle present distinct challenges for road damage detection, making it an ideal representation of real-world scenarios. The dataset includes four types of damage: longitudinal cracks, transverse cracks, crocodile cracks, and potholes, with sample sizes of 4049, 3979, 6199, and 2243, respectively. In comparison, the Indian dataset is more limited, primarily featuring road images under consistent conditions such as clear weather and daytime lighting. This lack of environmental variety reduces its ability to reflect the complexities of road damage in different settings. As such, its usefulness in training models for more varied conditions, like those in Japan, is somewhat constrained. Additionally, the Norwegian dataset offers a unique challenge, with images taken in snowy, rainy, and low-light conditions, making it a valuable resource for testing the performance of model in harsh weather. Future work will prioritize improving detection accuracy in these challenging environments, particularly in low-light and snowy conditions, to further adapt the model to real-world road detection needs across different climates.

To demonstrate the effectiveness of YOLO-RD in road damage detection, we evaluate our model using several metrics, including mAP, mAP_50_, mAP_*s*_, mAP_*m*_, and mAP_*l*_. Mean Average Precision (mAP) measures the overall detection accuracy across all classes. mAP_50_ calculates the precision at an IoU threshold of 0.5. The metrics mAP_*s*_, mAP_*m*_, and mAP_*l*_ assess detection precision for small, medium, and large objects, respectively, based on their area sizes: mAP_*s*_ for objects smaller than 32 × 32 pixels, mAP_*m*_ for those between 32 × 32 and 96 × 96 pixels, and mAP_*l*_ for objects larger than 96 × 96 pixels. Giga Floating Point Operations Per Second (GFLOPS) evaluates the computational efficiency of model, while Parametersindicates the total number of parameters in the model.

### 4.2. Implementation Details

The proposed model and comparison models were implemented using the MMYOLO and MMDetection frameworks. YOLO-RD was trained on two RTX 3090 GPUs for 200 epochs, while the other models were trained using their default configurations. Both MMYOLO and MMDetection employed the SGD optimizer with a learning rate of 0.02. By default, the batch size was set to 16, and the confidence threshold for visualization was set to 0.5. The input and test image sizes for MMDetection were both 1333 × 800 pixels, while for MMYOLO, the input image size was set to 640 × 640 pixels.

### 4.3. Ablation Studies

As shown in Table 1, the ablation study highlights the significant improvements achieved by integrating the following three modules into the YOLOv8-based model, demonstrating clear advantages over the baseline YOLOv8-s [21].

Effect of the Star Operation Module (SOM). The introduction of the SOM significantly improves the detection performance of model, particularly in detecting small objects, which are often a critical challenge in road damage detection. Specifically, the overall mAP increases from 23.71 to 25.03, with small object detection (mAP_*s*_) improving from 12.73 to 14.32, medium object detection (mAP_*m*_) rising from 19.83 to 21.02, and large object detection (mAP_*l*_) increasing from 27.71 to 29.22. This improvement is primarily due to the high-dimensional feature projection capability of the SOM, which allows the model to better capture small-scale features like road cracks that are often difficult to detect. The SOM works by applying depthwise convolutions and pointwise convolutions, combined with element-wise multiplication, to enhance both spatial and semantic representations of small objects. By projecting features into a higher-dimensional space, the SOM enables the model to focus more effectively on subtle road damage, such as small cracks, which are often overshadowed by larger objects or background noise. This makes the model more sensitive to fine details, improving its ability to detect small cracks, which is a critical task in road surface analysis. At the same time, the SOM introduces a moderate increase in computational cost, with Flops growing from 14.27 G to 18.21 G and the number of parameters (Parameters) increasing from 11.14 M to 16.33 M. Despite this slight increase in complexity, the SOM offers a significant improvement in detection accuracy. The ability of the SOM to enhance small object detection, especially road cracks, proves to be highly valuable for road damage detection, where accurate identification of even the smallest defects plays a crucial role in road safety and maintenance.

Effect of the Multi-dimensional Auxiliary Fusion (MAF). The integration of the Multi-dimensional Auxiliary Fusion (MAF) module results in a significant enhancement in the performance of model. Specifically, the overall mAP increases from 23.71 to 25.26, with mAP_*s*_ showing the most notable improvement, rising from 12.73 to 16.23. Additionally, mAP_*m*_ increases from 19.83 to 21.34, and mAP_*l*_ increases from 27.71 to 29.04. These improvements demonstrate MAF’s effectiveness in addressing the challenge of detecting small-scale objects like road cracks, especially in complex background scenarios where such features can be easily overlooked or overshadowed by larger objects or noise. MAF achieves this by aggregating multi-scale features and dynamically adjusting their contribution at different stages of the network. This fusion of low-level spatial features with high-level semantic features enables the model to retain critical details necessary for detecting small road damage while suppressing irrelevant background noise. The ability to adaptively prioritize important features, particularly those related to small cracks, results in significant improvements in mAP_*s*_. Regarding computational cost, the introduction of MAF leads to a moderate increase. Specifically, Flops rise from 14.27 G to 18.45 G, and the number of parameters increases from 11.14 M to 16.87 M. While this results in a slight increase in computational complexity, the trade-off between performance and complexity is well-balanced. The significant improvements in detection accuracy, particularly for small object detection, justify this increase. MAF’s ability to enhance model performance in challenging road crack detection scenarios makes it an essential component of YOLO-RD, significantly improving detection precision, especially in complex real-world environments.

Effect of the Wavelet Transform Convolution (WTC). The integration of the Wavelet Transform Convolution (WTC) module in the detection head results in notable improvements in the performance metrics of model. Specifically, the overall mAP increases from 23.71 to 24.04, with mAP_*s*_ showing the most significant improvement, rising from 12.73 to 16.35, highlighting WTC’s effectiveness in enhancing the detection of small objects, such as small road cracks. This improvement is attributed to the ability of module to decompose feature maps into distinct frequency components, which allows the model to focus on both fine details and larger structures, improving its detection capabilities across multiple scales. Meanwhile, mAP_*m*_ increases slightly from 19.83 to 20.16, and mAP_*l*_ remains stable at 27.76, suggesting that while WTC excels at small object detection, its impact on medium and large objects is more modest. In terms of computational cost, the introduction of WTC leads to a reduction in both Flops and the number of parameters, making the model more computationally efficient. Specifically, Flops decrease from 14.27 G to 10.45 G, and the number of parameters drops from 11.14 M to 9.37 M. This reduction demonstrates that the WTC module not only maintains detection accuracy but also enhances computational efficiency by focusing on essential frequency components, rather than processing the entire feature map. The reduced computational overhead allows for faster inference, which is particularly beneficial for real-time road damage detection in resource-constrained environments. Overall, the integration of WTC provides a practical solution to the challenges of detecting irregular cracks in road surfaces, particularly small, subtle cracks that are often difficult to detect. The most noticeable improvements are observed in small object detection, confirming that the computational efficiency and performance enhancements achieved by WTC are well suited for real-world applications, making it an essential component in the YOLO-RD framework.

### 4.4. Comparisons

Comparison Experiments. Our proposed method was compared with several mainstream object detection models, including single-stage detectors (YOLOv5-s, YOLOv7-tiny [44], YOLOX-s, YOLOv8-s, YOLOv10-n [27]) and two-stage detectors (Faster R-CNN [30], Cascade R-CNN [32]). As shown in Table 2, among single-stage detectors, our mAP is 25.75, while YOLOv8-s achieves 23.71. Additionally, our mAP_*s*_ is 17.66, whereas YOLOv8-s achieves 12.73. Although our model has a slight increase in computational cost, the significant improvements in mAP and mAP_*s*_ demonstrate the clear advantage of our method in overall performance and small object detection. Compared to YOLOv10-n, our mAP is 25.75, while YOLOv10-n achieves 25.31. Similarly, our mAP_*s*_ is 17.66, compared to YOLOv10-n’s 15.30. This indicates that our model not only outperforms YOLOv10-n in overall performance but also demonstrates superior performance in small object detection, with more balanced performance across objects of different scales. In comparison with the two-stage detector Cascade R-CNN, our mAP is 25.75, while Cascade R-CNN achieves 25.03. For mAP_*s*_, our model achieves 17.66, whereas Cascade R-CNN achieves only 12.82. Additionally, in terms of computational efficiency, our model has 20.89M parameters, which is significantly lower than the 69.16M parameters of Cascade R-CNN. This demonstrates that our method achieves comparable detection accuracy while being much more efficient, making it particularly suitable for practical deployment. These results indicate that our proposed method achieves an excellent balance between accuracy and efficiency, making it especially well suited for road crack detection tasks in complex and visually challenging environments. It can detect small, low-contrast cracks and subtle damage with high precision, ensuring reliable performance and establishing itself as a robust and practical solution for real-world road damage detection applications.

### 4.5. Visualization

**Figure 7:**
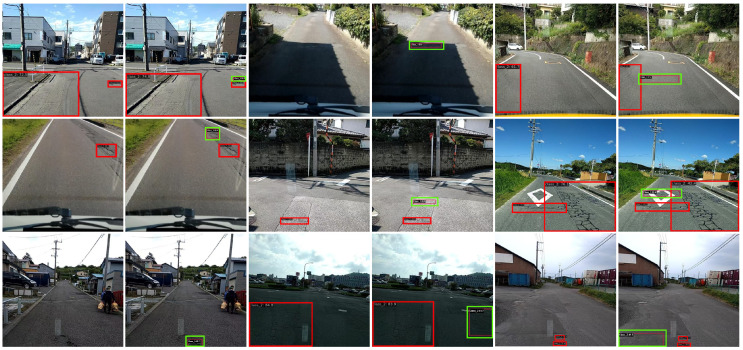
Visualization of YOLOv8 (**left**) and YOLO-RD (**right**) detection results.

As shown in Figure 7, the comparison between YOLOv8 (left) and the proposed YOLO-RD (right) highlights significant improvements in road damage detection. Three scenarios are analyzed: small damage detection (Left Group), complex background detection (Middle Group), and multi-scale damage detection (Right Group). Red boxes represent actual detected results, while green boxes indicate discrepancies between the two models.

The red boxes in Figure 7 represent road damage that can be detected by the YOLOv8 model, and the green boxes represent road damage that cannot be detected by YOLOv8 but can be detected by YOLO-RD. Note that the road damage marked by the red box can also be detected by YOLO-RD.

In the first row of the leftmost group, the cracks are thin and faint, making them difficult to detect due to their low visibility. YOLOv8 fails to recognize the full extent of the crack, as the red box shows partial detection. In contrast, YOLO-RD successfully captures the entire crack, as indicated by the complete bounding box (green), demonstrating its superior ability to detect low-contrast, small-scale cracks that are often overlooked. In the third row of the middle group, cracks are obscured by shadows cast across the road surface, significantly reducing their visual contrast. YOLOv8 struggles to distinguish the damages from the darkened regions, leading to missed detections or incomplete bounding boxes. In contrast, YOLO-RD accurately detects the cracks within the shadowed areas, as highlighted by the green boxes. This demonstrates the robustness of YOLO-RD in handling low-contrast conditions caused by environmental shadows, ensuring reliable detection even under visually challenging circumstances. In the first row of the rightmost group, cracks appear at varying scales, including wide, prominent fissures and thin lines. YOLOv8 detects the larger fissure but fails to identify the smaller crack, as shown by the absence of bounding boxes. YOLO-RD, on the other hand, successfully detects cracks of multiple scales, providing precise and tight bounding boxes. This highlights the capability of YOLO-RD to adapt to multi-scale variations, ensuring comprehensive detection.

The visualization results in Figure 6 demonstrate the clear advantages of YOLO-RD over YOLOv8 for road damage detection. Specifically, YOLO-RD excels in detecting fine cracks, resolving low-contrast interferences caused by shadows, and accurately identifying cracks of varying scales. These advancements position YOLO-RD as a robust and reliable solution for automated road condition monitoring, enabling accurate detection under diverse real-world conditions.

## 5. Discussion

This study introduces YOLO-RD, an innovative framework designed to address key challenges in road damage detection, such as small-scale damages, complex backgrounds, and irregularly shaped features. By integrating three core innovations—the Star Operation Module (SOM), Multi-dimensional Auxiliary Fusion (MAF), and Wavelet Transform Convolution (WTC)—YOLO-RD significantly enhances its capability to detect diverse road damages in challenging real-world conditions. These modules collectively improve the model’s ability to detect subtle, low-contrast, and multi-scale damages, as confirmed by extensive experiments. The model consistently outperforms baseline methods across critical metrics, including mAP and mAP_*s*_, demonstrating its potential as a reliable solution for automated road condition monitoring.

Despite the substantial success of YOLO-RD, several areas remain for further improvement. Future work will focus on the following directions:

1. Extending Data Collection and Analysis: To improve the generalization of the model, we will expand the dataset to include a wider range of damage types typical for both cement concrete and asphalt concrete pavements, addressing diverse geographical and material conditions. Specifically, we will collect data from different climatic regions and road types to include a broader variety of damage patterns and environmental contexts.

2. Adapting to Extreme Weather and Lighting Conditions: To increase the robustness of the model under harsh weather and low-light conditions, we plan to enhance the model’s performance in scenarios such as heavy rain, snowfall, and dim lighting, where road damage features become more difficult to detect. We will augment the dataset with images from these challenging environments and adjust the model’s training strategy to improve its capability to recognize low-contrast damage features, using techniques such as data augmentation to simulate rain and snow effects and improve low-light performance.

3. Improving Computational Efficiency: We aim to reduce computational complexity while maintaining model performance. This will involve applying various techniques, such as the following:

Model Pruning: Removing unnecessary connections or neurons to reduce computation and storage, enhancing inference speed.

Quantization: Converting floating-point computations to lower precision to reduce the computational load and memory usage.

Lightweight Design: Replacing the current architecture with more efficient models such as MobileNet or EfficientNet, ensuring high performance on resource-constrained devices without compromising detection accuracy.

These approaches will allow YOLO-RD to be deployed in real-time systems and on devices with limited computational resources, such as mobile devices and drones, without sacrificing performance.

4. Integrating Multi-Task Learning: Expanding the scope of YOLO-RD by integrating multi-task learning to handle additional road-related tasks, such as the following:

Crack Severity Assessment: Evaluating the severity of detected cracks based on features like width and depth to assess their potential impact on the road structure.

Road Type Classification: Incorporating a task to classify the road type (e.g., asphalt, concrete), enabling customized road maintenance strategies based on road material.

Repair Strategy Recommendation: Leveraging the detection results to automatically generate repair priority and maintenance strategies, facilitating more intelligent and effective road maintenance decisions.

Additionally, a key limitation of the current model is its performance in detecting extremely small cracks, particularly in low-contrast environments or when covered by significant background noise. Future improvements will focus on the following:

Enhancing Small Crack Detection: Adopting finer feature extraction techniques, such as local feature extraction and multi-scale convolutions, to improve the detection of microcracks that are often overlooked by current models.

Background Noise Suppression: Further optimizing the model’s performance in complex backgrounds by incorporating background noise suppression techniques or region-of-interest (ROI) extraction, ensuring that the model can better isolate and identify small cracks.

By addressing these areas, YOLO-RD can further enhance its performance and applicability in diverse real-world scenarios, making significant contributions to road safety and efficient pavement maintenance. This will result in a more reliable, efficient, and practical solution for automated road crack detection, even under challenging conditions.

## Figures and Tables

**Table 1:** Ablation experiments.

Models	mAP	mAP_50_	mAP_*s*_	mAP_*m*_	mAP_*l*_	Flops/G	Parameters/M
YOLOv8-s (baseline)	23.71	52.92	12.73	19.83	27.71	14.27	11.14
YOLOv8-s+SOM	25.03	55.24	14.32	21.02	29.22	18.21	16.33
YOLOv8-s+MAF	25.26	55.47	16.23	21.34	29.04	20.16	17.46
YOLOv8-s+WTC	24.04	53.66	16.35	20.16	27.76	10.45	9.37
YOLOv8-s+SOM+MAF	25.65	55.53	16.27	21.31	29.54	24.10	22.65
YOLOv8-s+MAF+WTC	24.68	54.96	16.28	21.35	30.07	16.33	15.70
YOLOv8-s+SOM+WTC	25.10	55.32	16.42	21.19	29.38	14.39	14.57
YOLOv8-s+SOM+MAF+WTC	25.75	55.62	17.66	21.37	29.58	20.28	20.89

**Table 2:** Comparison experiments.

Models	Backbone	mAP	mAP_50_	mAP_*s*_	mAP_*m*_	mAP_*l*_	Flops/G	Parameters/M
YOLOv5-s	YOLOv5CSPDarknet	18.23	44.02	10.25	15.61	19.93	8.16	7.15
YOLOv7-tiny	E-ELAN	13.51	36.21	6.92	11.36	15.22	6.78	6.15
YOLO X-s	YOLOXCSPDarknet	19.43	46.15	10.86	16.02	21.47	13.54	9.06
YOLOv8-s	YOLOv8CSPDarknet	23.71	52.92	12.73	19.83	27.71	14.27	11.14
YOLOv10-n	CSPDarknet	25.1	53.7	13.78	20.14	29.53	8.25	2.69
Faster R-CNN	ResNet-50	22.01	52.1	9.8	17.9	28.1	101.00	28.29
Cascade R-CNN	ResNet-50	25.03	54.95	12.82	21.16	31.44	162.00	69.16
Ours	CSPDarknet	25.75	55.62	17.66	21.37	29.58	20.28	20.89

## Data Availability

The dataset used in this study, RDD2022, is publicly available at the following GitHub repository: https://github.com/sekilab/RoadDamageDetector.
